# Postoperative challenges addressed through nursing care of patients receiving lower extremity tumor prosthesis

**DOI:** 10.1186/s12912-024-02400-2

**Published:** 2024-10-04

**Authors:** Marina Golemac, Müjgan Yilmaz, Michael Mørk Petersen

**Affiliations:** Rigshospitalet, Department of Orthopedic Surgery, University of Copenhagen, Inge Lehmanns Vej 6, 2100, Copenhagen, 35450640 Denmark

**Keywords:** Nursing care, Postoperative complications, Patient outcome, Bone sarcoma, Orthopedic surgery, Tumor prostheses

## Abstract

**Background:**

Patients with primary Bone Sarcoma and Giant Cell Tumors in the lower extremities often require major surgery involving tumor prostheses. The postoperative course for this patient group can be complex and influenced by various factors and challenges that demand careful nursing care. This study aims to identify challenges related to the nursing care of individuals with primary bone tumors following surgery for tumor prostheses in the lower extremities.

**Methods:**

A retrospective cohort study of 15 patients treated at Rigshospitalet, Copenhagen, Denmark, between November 5. 2016, and April 1. 2020 was conducted by medical record review, focusing on challenges related to postoperative nursing care. All patients with the surgery code “Bone Excision” were identified within the surgery booking system and screened for eligibility.

**Results:**

Patients experienced postoperative challenges such as severe pain, prolonged time to mobilization (mean: 4 days), and defecation (mean: 5 days). The mean length of stay at the Rigshospitalet was 13 days. Furthermore, eleven patients (73%) reported disrupted sleep and nausea.

**Conclusion:**

Patients undergoing tumor prosthesis surgery in the lower extremities face considerable postoperative challenges that contribute to a prolonged hospital stay. These challenges, including severe pain, delayed mobilization, and gastrointestinal issues, significantly impact recovery. The findings highlight the urgent need for targeted nursing interventions to address these issues effectively. Enhanced pain management protocols, early mobilization strategies, and comprehensive postoperative care plans are essential to improve patient outcomes and reduce the length of hospital stays. Addressing these challenges through dedicated nursing care is crucial for optimizing the recovery process for patients receiving lower extremity tumor prostheses.

## Introduction and background

Primary Bone Sarcoma (BS) is a severe disease, with an average incidence of 63 new cases registered annually in Denmark between 2012 and 2016, with a slightly higher male predominance [1]. Sarcoma is a type of cancer developing from connective or supporting tissue [2]. The most prevalent BS types are Osteosarcoma, Ewing sarcoma, and Chondrosarcoma. Giant Cell Tumors of bone (GCT) are benign, but locally aggressive tumors that are sometimes treated surgically the same way as BS. In this study, “primary bone tumors” collectively refer to both BS and GCT [3]. The treatment of Osteosarcoma and Ewing sarcoma involves a combination of surgery and chemotherapy while for Chondrosarcoma and GCT, the treatment is usually only surgical. The overall five-year survival prognosis for BS is approximately 60–70% [3]. The main purpose of primary bone tumor surgery is often to achieve complete resection of the tumor. In the past, the main approach to achieve this was through radical resection, which often involved amputation of the affected limb. The use of tumor prostheses has increased in the last 30 years and is now the preferred method of reconstruction following segmental resection of long bones in the extremities [4]. This is due to their widespread availability, ease of use, and the ability to preserve the affected limb [5]. However, despite the widespread adoption of tumor prostheses, relatively little is known about the specific nursing challenges encountered during the postoperative care of these patients. In orthopedic surgical wards, oncology patients represent a minority of the total patient group, and sarcomas account for approximately 0.8% of all cancers [3]. Despite their minority status, oncology patients in orthopedic surgical wards present significant complexity and demand due to the complexities of their diagnoses, treatments, and multidisciplinary care requirements. Orthopedic nurses play a vital role in the comprehensive care of patients after surgery of the lower extremity with tumor prostheses due to primary bone tumors. These patients encounter numerous postoperative challenges, which can significantly influence their overall recovery [6]. To the best of our knowledge, this study is the first to address the challenges in orthopedic nursing in patients with lower extremity prostheses due to primary bone tumors. Therefore, this retrospective study aims to provide an overview of the challenges faced in the early postoperative course by patients, who have undergone tumor prosthesis implantation for a primary bone tumor. This study will elucidate which elements are important for the postoperative course with a focus on orthopedic nursing to initiate a targeted preventive effort in the future.

## Materials and methods

### Study design and eligibility criteria

**Fig. 1:**
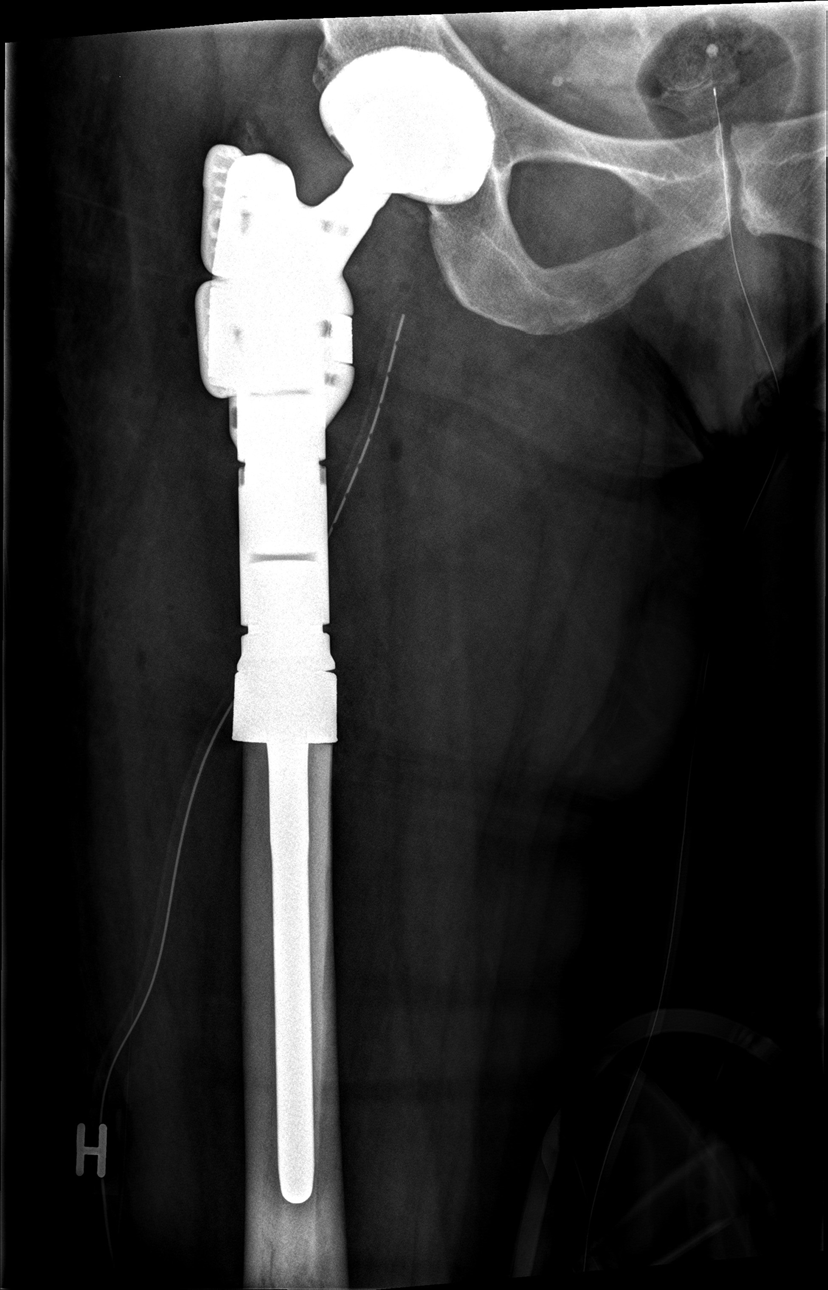
Mega tumor prosthesis hip

**Fig. 2:**
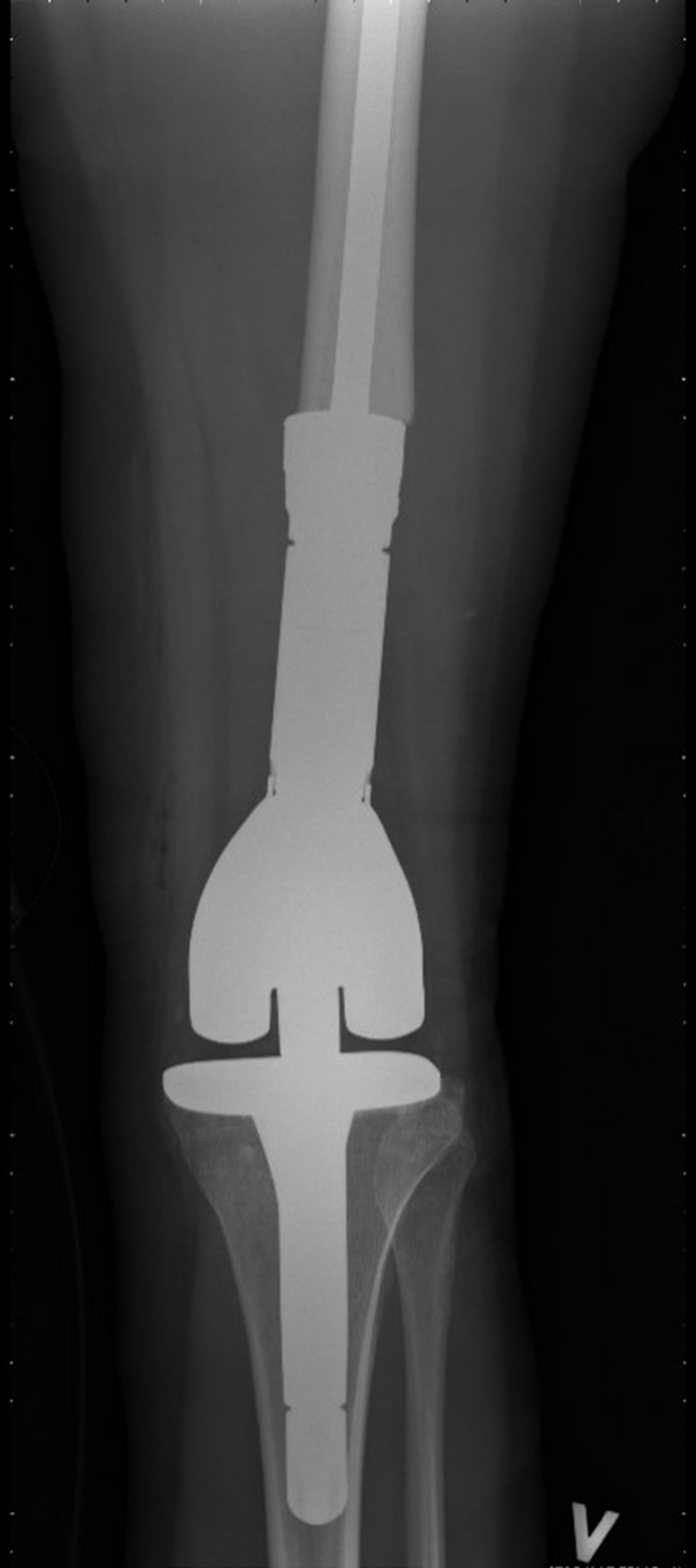
Mega tumor prosthesis knee anteroposterior

**Fig. 3:**
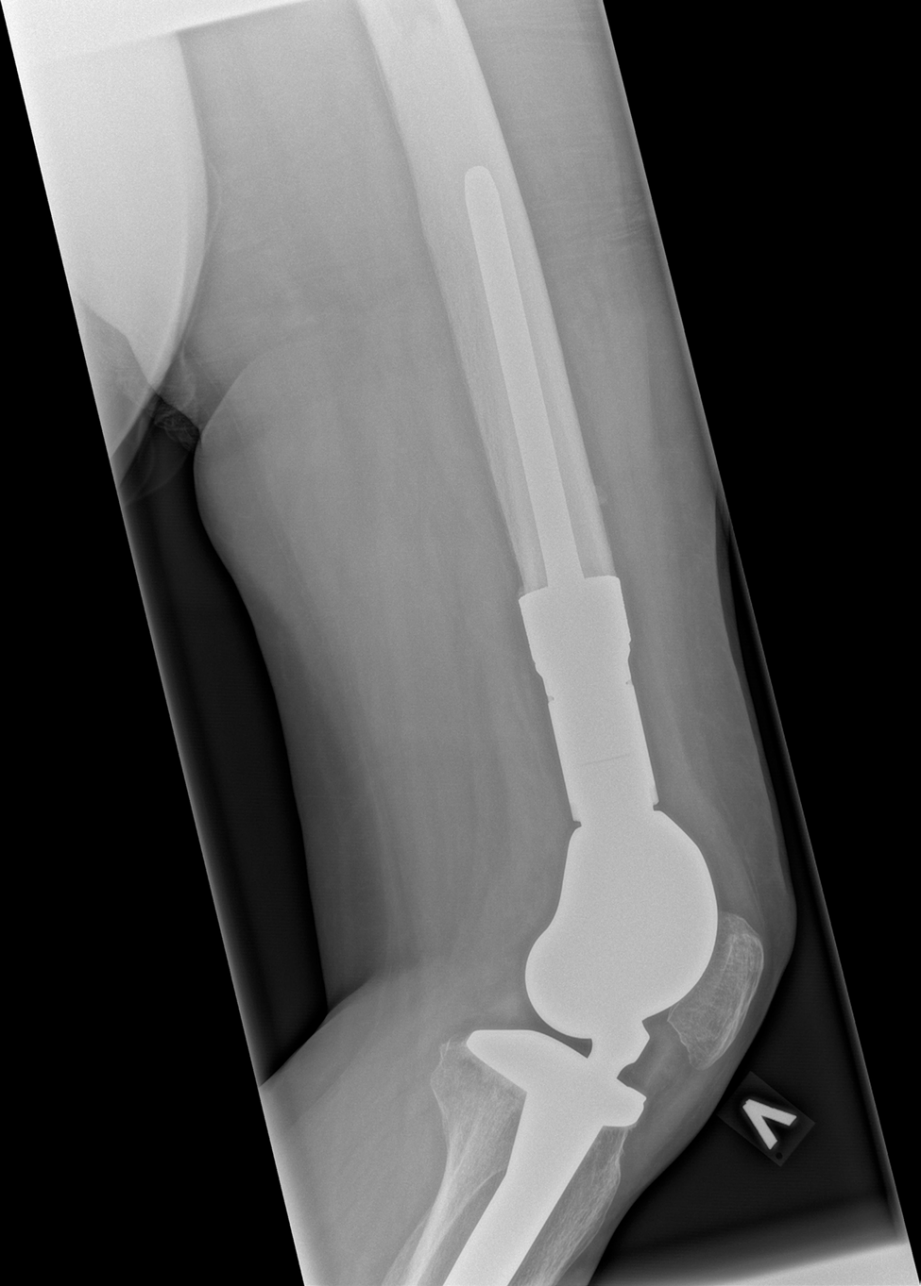
Mega tumor prosthesis knee lateral

**Fig. 4:**
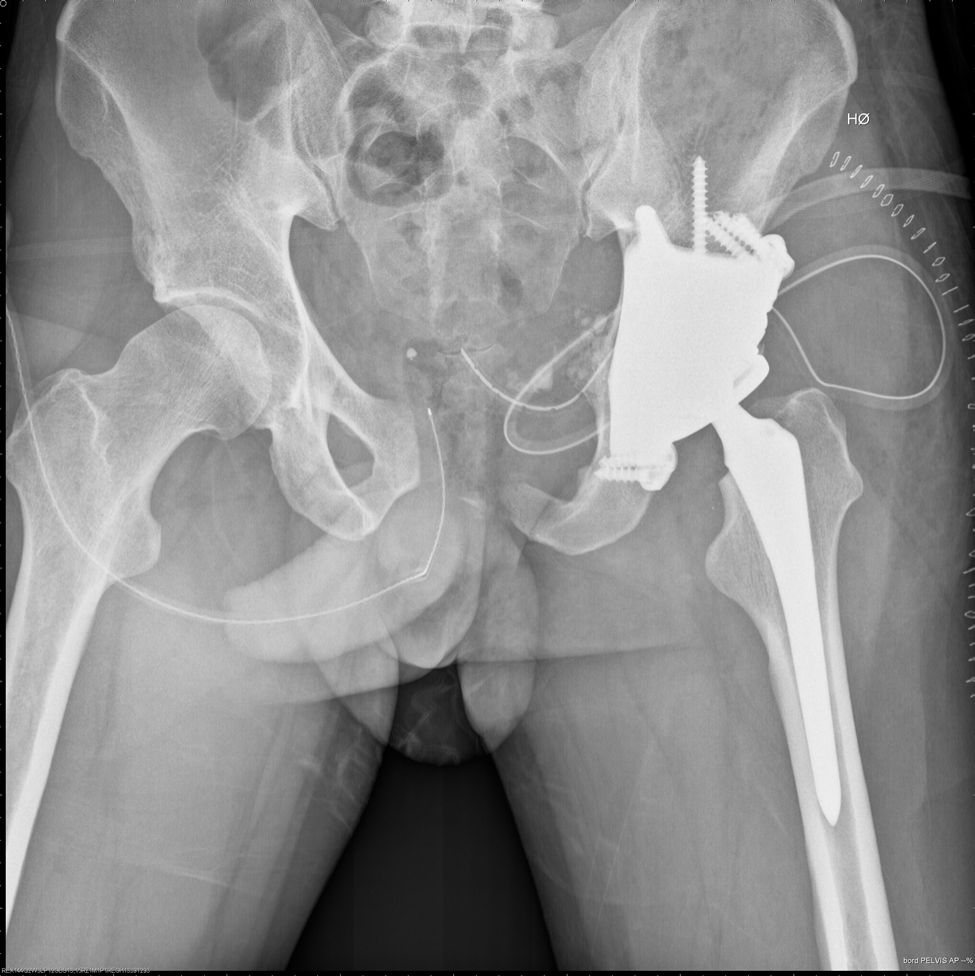
Mega tumor prosthesis pelvis

This study is a retrospective cohort of patients receiving a tumor prosthesis due to treatment of primary bone tumors of the lower extremities at the Department of Orthopedic Surgery, Musculoskeletal Tumor Section, Rigshospitalet, Copenhagen, Denmark. We included patients with surgery between November 2016 and April 2020 who met the following inclusion criteria: Age ≥ 18 years, diagnosed with a primary bone tumor, and having undergone bone resection and reconstruction with a tumor prosthesis in the pelvis, hip, or knee joint (Figs. 1, 2, 3 and 4). We excluded patients with revision surgery of tumor prosthesis, surgery for metastasis of another primary cancer, and patients diagnosed with a primary bone tumor without tumor prosthesis surgery.

### Enrollment and data collection

Patients were identified within the surgery booking system, *Snapbord* (SB), part of the hospital´s administrative system, and the electronic patient record system *Sundhedsplatformen* (SP). The SB was reviewed daily. All patients with the surgery code “Bone excision” were screened for eligibility, which included a systematic review of their electronic patient record for inclusion and exclusion criteria. The following variables were collected from medical records: Length of stay (LOS), vital status (deceased or alive), cancer diagnosis (pathology), gender, weight, blood transfusion, anatomical location of the prosthesis, prosthesis side, revision status, whether chemotherapy was administered before and after surgery, post-operative pain, pain medications, and pain intensity rated on the Numerical Rating Scale (NRS) ranging from 0 to 10 (0 = none, 1–3 = mild, 4–6 = moderate, 7–10 = severe) [7]. We also registered the following: Wound leakage and signs of wound infection, microbiology results, type and duration of antibiotics administered during hospitalization, consultations requested from other specialties, the time to the first postoperative out-of-bed mobilization, assessment of sleep quality by nurses, the timing of first postoperative defecation, presence of a drain, occurrence of nausea and administration of antiemetics were registered. Leakage was defined as a medical record note indicating wound leakage, a visible leakage observed on the plaster, or if daily plaster changes were documented. Nausea was defined as a medical record note indicating that the patient experienced nausea, vomiting, or anti-emetic medications were administered as needed (PN– pro necessitate). Out-of-bed mobilization was defined as a medical record note, typically made by the physiotherapist, or if documented in assessment forms during movement and activity, specifically mobilization or out-of-bed activities.

### Statistical analysis and ethical statements

Results are presented using descriptive statistics, including percentage, mean, and range. Approval from the Danish Data Protection Agency (project nr.: P-2020-603) and the Patient Safety Authority (j.nr.31-1521-372) was obtained. The data were organized and analyzed using Microsoft Excel.

## Results

### Patients

We included 16 patients (11 females and 5 males) with a mean age of 41 years (range: 18–72). Bone resection localization was as follows: proximal femur (*n* = 3), pelvis (*n* = 2), distal femur (*n* = 5), total femur (*n* = 2), proximal tibia (*n* = 3) and proximal tibia and distal femur (*n* = 1). Eight patients had prostheses inserted on the right side and eight on the left. Patients had primary surgery due to the following diagnoses: Ewing sarcoma (*n* = 4), osteosarcoma (*n* = 5), chondrosarcoma (*n* = 4), GCT (*n* = 2), and myxofibrosarcoma (*n* = 1). One patient was amputated the day after primary surgery due to arterial thrombosis, resulting in 15 patients available for follow-up (Table 1).

**Table 1:** Demographics

	All (*n* = 16)	Female (*n* = 11)	Male (*n* = 5)
**Mean age at time of surgery (range)**	41 (18–72)	46 (19–72)	29 (18–49)
**Weight in kg. (range)**	71 (49–133)	65 (49–95)	85 (63–133)
**Mean high in cm (range)**	170 (158–185)	168 (158–174)	176 (167–185)
**Mean MBI in kg m2 (range)**	24 (19–39)	23 (19–34)	27 (22–39)
**Gender**	16	11	5
***Pathology***			
**Ewing sarcoma**	4	3	1
**Osteosarcoma**	5	3	2
**Chondrosarcoma**	4	3	1
**Giant cell tumor**	2	1	1
**Myxofibrosarcoma**	1	1	0
***Bone resection location***			
**Proximal femur**	3	3	0
**Distal femur**	5	3	2
**Total femur**	2	2	0
**Proximal tibia**	3	2	1
**Proximal tibia + distal femur**	1	1	0
Pelvis	2	0	2

### Clinical outcome

When data analysis was finalized, 3 patients were deceased, on average 10 months after primary surgery (range: 2–17 months).

### Hospitalization and surgery

The mean duration of stay at Rigshospitalet was 13 days (range: 5–32). Ten patients were discharged directly to their homes, while 5 patients were transferred to a local regional hospital due to the need for further mobilization and pain management issues.

### Blood transfusion

Of the total sample size (*n* = 15), ten patients required blood transfusion peri-and/or postoperatively. Specifically, these transfusions consisted of packed red blood cells (SAG-M) (*n* = 78), fresh frozen plasma (*n* = 36), thrombocytes (*n* = 10), and human albumin (*n* = 3).

### Antibiotics

All patients (*n* = 15) were treated with Cefuroxime 1.5 g x 3 postoperatively until the surgical wound was dry, with a mean treatment duration of 8 days (range: 4–22). However, in three cases, additional narrow-spectrum antibiotic therapy after the termination of Cefuroxime was necessary to address specific infections.

### Mobilization

The time to the first postoperative out-of-bed mobilization was 4 days (range: 2–4). Data was available for all 15 patients.

### Defecation

Data on postoperative bowel movement was available for 12 patients. The time to first postoperative defecation was 5 days (range: 2–7). One patient whose first documented defecation was on day 17, with no medical record indicating a gastrointestinal malfunction, was excluded from the primary analysis.

### Nausea

Out of 15 patients available for follow-up, 11 patients experienced nausea. Additionally, two patients were treated with antiemetics. However, there is missing data for one patient, and there is no documentation regarding whether antiemetics was administrated to another patient who experienced nausea. Among the patients who received antiemetics, the range of the number of times antiemetics were administered was 1 to 24.

### Sleep

The included patients (*n* = 15) experienced variable quality of sleep as documented in SP by the night nurse. The documentation indicated the following patterns: sleeping during night round (*n* = 4), single night disturbed sleep (*n* = 5), every night disturbed sleep (*n* = 1), several times disturbed sleep (*n* = 4), and a couple of times disturbed sleep (*n* = 1).

### Pain

NRS values were registered in 53% of patients on day 0 and day 1 and 20% of patients on day 2 (Table 2). When NRS values had been registered, it was only rarely specified whether patients were at rest or active. The mean NRS value during the hospital admission was 3 (range: 0–8), but this data has a high degree of uncertainty due to the lack of documentation [8]. Despite having the epidural catheter (EPI), 33% of patients required additional consultation from an anesthesiologist, while 53% of patients needed medication more than 5 times a day in addition to planned medication.

**Table 2:** Main findings

	In total (*n* = 15)	Female (*n* = 10)	Male (*n* = 5)
**Mean time in days to first postoperative defecation (range) [number of patients documented]**	5 (2–7) [*n* = 12]	5 (2–7) [*n* = 9]	5 (5) [*n* = 3]
**Nausea registered by nurse (percentage)**	*n* = 11 (73%)	*n* = 6 (40%)	*n* = 5 (33%)
**Hospitalization - mean time in days (range) [number of patients documented]**	13 (5–32) [*n* = 15]	13 (5–24) [*n* = 10]	14 (6–32) [*n* = 5]
**First out-of-bed mobilization postoperative in mean days (range) [number of patients documented]**	3 (2–4) [*n* = 15]	3 (2–4) [*n* = 10]	3 (2–4) [*n* = 5]
**Pain: consult from anesthesiologist (percentage)**	*n* = 5 (33%)	*n* = 2 (13%)	*n* = 3 (20%)
**Interrupted sleep (percentage)**	*n* = 11 (73%)	*n* = 7 (47%)	*n* = 4 (27%)

The main findings during the review of the 15 journals are presented in Table 3.

**Table 3:** NRS

Patient number	Post-operative day 0	Post-operative day 1	Post-operative day 2
**1**	ES	ES	ES
**2**	ND	4–6	3–6
**3**	ND	ND	ND
**4**	2	0–3	Moderate pain (4–6) (not from operation area)
**5**	ND	ND	Acute pain
**6**	3–8	Pain registered (not from operation area)	ND
**7**	0	ND	ND
**8**	ND	ND	Acceptable, no complaints
**9**	Mild pain (1–3)	0	2–3
**10**	0–8	God effect of painkillers	0–6(no pain during rest, some pain during activity)
**11**	ND	4–6	4–6
**12**	ND	0	ND
**13**	0	0	0–5
**14**	0	1–8	5–6
**15**	0	0–2	Good effect of painkillers
**16**	5–8	4–6	ND

Measured in the first two postoperative days in patients with a tumor prosthesis in the lower extremities. (Not Documented = ND) (Excluded from study = ES)

## Discussion

This retrospective study aimed to provide an overview of the challenges faced in the early postoperative course by patients, who have undergone tumor prosthesis implantation for a primary bone tumor. The main findings identified major postoperative nursing (and patients) challenges, such as high LOS, delayed defecation, nausea, pain management, delayed mobilization, and sleep disturbances. The average LOS was 13 days. One-third of the patients were transferred to a regional hospital due to further mobilization and pain management issues, so the total duration of their hospitalization is unknown. The need to transfer one-third of patients to a regional hospital suggests that this group of patients is more complex despite having the same postoperative complications in comparison with other major surgeries. To our knowledge, there is no published data on the LOS specifically for this group of patients. In Denmark, a nationwide study conducted in 2010 found that the average LOS after THA and TKA was 7.4 and 8 days, respectively [9]. However, there has been an increase in the utilization of day-case surgery from 2010 to 2020. A Danish nationwide register study examining the 10-year evolution of day-case procedures for THA and TKA reveals that the mean LOS for THA has decreased to 2.3 days, while for TKA, it has decreased to 2.6 days [10]. In contrast with the fact that in 2020, we continue to observe an average LOS of 13 days for our patients, even though they largely experience similar types of challenges. A recent study conducted across eight arthroplasty centers in Denmark investigated the feasibility of same-day discharge following primary hip and knee arthroplasty. The prospective multicenter cohort study, covering 40% of the annual hip and knee arthroplasties in Denmark, demonstrated that outpatient surgery was achievable in 21% of patients, with the potential for increased rates as centers gain more experience [11]. It suggests a relatively long and complex recovery process associated with our patient group and the procedure being performed, yet the reasons for this complexity remain unclear. One of the main findings is the delayed time of first defecation postoperatively, with a mean of 5 days. Constipation is often characterized by the infrequent occurrence of bowel movements, typically less than three times a week [12]. On the one hand, this study´s findings reveal a delayed time of first defecation postoperatively, indicating a potential issue with gastrointestinal dysfunction, specifically constipation. This aligns with the Danish study, which reported a high proportion of patients (69.1%) experiencing constipation after hip fracture surgery [13]. These findings suggest that constipation is a common problem among postoperative patients and should be addressed by healthcare providers. On the other hand, the missing documentation of the defecation status for 20% of patients in this study introduces uncertainty regarding the timing of the first postoperative defecation. The nurse has a major role and must provide strong supportive communication while assisting with defecation which has important significance on patients´ quality of life [14]. The surgical stress response, anesthesia, missing bowel management protocols (including the use of laxative and stool softeners), and changes in routine such as using a bedpan, inadequate privacy, and not being able to have a proper toilet posture can contribute to suppressing the urge to defecate, gastrointestinal dysfunction, including constipation [15–18]. Additionally, opioid treatment and delayed mobilization can further exacerbate these issues [17, 19]. Our study emphasizes the need for better bowel management protocols. Preoperative education, abdominal massage, and appropriate use of laxatives and stool softeners are effective in reducing constipation rates [20]. Post-operative nausea and vomiting (PONV) was reported by 73% of patients in our study, with one patient´s data still missing. In comparison, a Swedish study focusing on patients undergoing Total Hip Arthroplasty (THA) and Total Knee Arthroplasty (TKA) under spinal anesthesia reported a lower incidence of PONV, with 46% of patients encountering this discomfort [21]. This notable discrepancy raises pertinent questions about the contributing factors behind the varying prevalence of PONV between our and the Swedish study [21]. Several factors contribute to PONV, including constipation, low blood percentage, opioid treatment, anesthesia, and the surgical procedure itself. The duration of the surgery and the required anesthesia duration are in our study results, demonstrating a mean surgery time of 301 min. Despite the numerous side effects associated with opioids, they are still widely used for postoperative pain management [22]. Our study indicates that patients in this group often require frequent pain medication with opioids, which could be a contributing factor to the high incidence of PONV. It is important to note that patients rank PONV as the most undesirable outcome, even more so than postoperative pain [23]. Overall, PONV causes stress for patients and leads to increased resource utilization, including longer recovery times, extended hospital stays, unexpected admissions, and healthcare costs [23]. The mean NRS value in our study during hospital stay was 3, a Portuguese study measured an average NRS 48 h postoperatively, with reported NRS 3.39 for THA and NRS 4.19 for TKA [24]. The similarity in NRS scores between our study and the Portuguese prospective study suggests that pain experiences among patients undergoing THA and TKA because of osteoarthritis during the initial postoperative period are consistent across different geographical regions and healthcare settings. Insufficiently treated pain can lead to PONV as well as decreased physical activity, which may result in prolonged bed rest and associated complications such as venous thrombosis and pulmonary embolism [25]. Early postoperative mobilization is essential for early functional recovery, but it can be inhibited by pain, paradoxically, it might also be limited by epidural pain management and opioids. One of the main reasons for delayed mobilization was epidural pain management, which reduced sensitivity and subsequently affected mobility [26]. Among others, one study has shown that early mobilization is associated with better mobility, prevention of complications such as constipation, and faster hospital discharges [27]. In our study, the average time to first mobilize out of bed was 3 days. A New York study with 900 patients undergoing TKA and THA divided them into two groups: one adhering to standard rehabilitation, mobilized on the first postoperative day, which was consistent with our study, and another receiving rapid rehabilitation, mobilized immediately in the recovery room. The study demonstrated a reduction in LOS for patients who received rapid rehabilitation. The average LOS for the standard rehabilitation group was 4.4 days, while for the rapid rehabilitation group, it was 3.9 days [28]. The goal was to mobilize the patient as soon as possible, aiming for postoperative day 0 or day 1 [27]. Enhanced Recovery after Surgery (ERAS), a concept invented in Denmark and implanted all over the world, aims to minimize postoperative complications, reduce LOS, improve pain management and accelerate the recovery process [27]. Delayed mobilization out of the bed could be one of the reasons for the longer hospitalization, which in this case was 13 days. Eleven patients (73%) in our study experienced interrupted sleep during their hospital stay. An observational cohort study from Bristol, including TKA and THA patients reported that between 44 and 57% of TKA and 21-52% of THA patients experienced disrupted sleep due to acute postoperative pain during their first three nights after surgery [29]. Effective pain treatment not only improves sleep but also enhances the overall quality of life [30]. It is well-known that sleep disorders can significantly impact patients’ quality of life [31, 32]. It is important to take into account that sleep quality can be influenced by various factors, such as pain, discomfort, medications, and the change of habit environment [33].

### Strengths and limitations

To our knowledge, this is the first study to investigate the challenges in orthopedic nursing of patients receiving lower extremities tumor prostheses. All patients who met the inclusion criteria in the study period were enrolled. The study´s strength lies in the fact that the BS surgery is performed at only two centers, making the sample size representative of the country´s population. However, the study does have several limitations. The most significant limitation of this study is the small population and limited sample size, which can potentially impact the findings and make it difficult to draw definitive conclusions. Additionally, the heterogeneity of diagnoses, tumor locations, and the variety of endoprostheses used introduces variability that may affect postoperative outcomes. The study’s retrospective nature also brings inherent limitations such as selection bias, data quality, and low precision of confidence. Furthermore, the clinical approaches described, including the post-operative antibiotic regimen, reflect practices specific to our institution, and the long duration of postoperative antibiotic therapy is not uncommon after surgery in orthopedic oncology patients receiving tumor prostheses. Recently, a randomized multicenter trial evaluated this practice [34]. This antibiotic regimen may not be universally adopted, potentially limiting the generalizability of our findings.

Missing data further increases the risk of selective reporting. It is crucial to be aware of these potential biases and consider them when interpreting the results. Therefore, it is important to exercise caution and promote further research before drawing conclusions based on these findings.

### Conclusion and relevance to clinical practice

Despite these limitations, our study contributes valuable preliminary data to an under-researched area, highlighting critical challenges in orthopedic nursing for patients with lower extremity tumor prostheses. The diversity in prosthesis types and tumor locations, while introducing variability, reflects real-world clinical scenarios and provides a comprehensive view of the complexities involved in such cases. Future studies should focus on larger, more homogeneous cohorts to better delineate these challenges and explore standardized approaches to postoperative care, and due to the rarity of these cancer diseases, further studies should probably be conducted as multicenter studies. Comparative analyses with more common surgical procedures, such as primary hip and knee arthroplasties, may also offer insights into optimizing care for this unique patient population.

We found that the major nursing challenges in patients who have undergone tumor prosthesis implantation for primary bone tumors are constipation, prolonged time to first post-operative mobilization, increased pain, nausea, and a significantly extended LOS. These challenges are deeply interrelated, creating a cycle of postoperative complications that hinder recovery. The results of this study emphasize the critical need for specific nursing interventions to disrupt this harmful cycle. By effectively managing these complications, we can significantly decrease the LOS and improve patient outcomes overall. Our findings can form the basis for future prospective qualitative studies evaluating interventions that can disrupt this detrimental cycle of postoperative complications and ultimately enhance patient outcomes.

## Data Availability

The data that support the findings of this study are available from corresponding author on reasonable request.

## Abbreviations

BS: Bone Sarcomas
ERAS: Enhanced recovery after surgery
EPI: Epidural
GCT: Giant cell tumors
LOS: Length of say
NRS: Numerical rating scale
PONV: Postoperative nausea and vomiting
PN: Pro necessitate
SB: Snapbord
SP: Sundhedsplatformen
THA: Total hip arthroplasty
TKA: Total knee arthroplasty

## References

[1] Kræftens Bekæmpelse [Internet]. Statistik om knoglesarkomer [cited 2023 Oct 12]; https://www.cancer.dk/knoglesarkomer-primaer-knoglekraeft-osteosarcom/knoglesarkomer-i-tal/

[2] Whelan JS, Davis LE. Osteosarcoma, Chondrosarcoma, and Chordoma. J Clin Oncol. 2018;36:188–93. 29220289 10.1200/JCO.2017.75.1743

[3] Sarkomer [Internet]. [cited 2023 Oct 10]; https://www.sundhed.dk/borger/patienthaandbogen/knogler-muskler-og-led/sygdomme/oevrige-sygdomme/sarkomer/

[4] Pala E, Trovarelli G, Calabrò T, Angelini A, Abati CN, Ruggieri P. Survival of modern knee tumor megaprostheses: failures, functional results, and a comparative statistical analysis. Clin Orthop Relat Res. 2015;473:891–9. 24874116 10.1007/s11999-014-3699-2 PMC4317408

[5] Pala E, Trovarelli G, Angelini A, Maraldi M, Berizzi A, Ruggieri P. Megaprosthesis of the knee in tumor and revision surgery. Acta Biomed. 2017;88:129–38. 28657574 10.23750/abm.v88i2-S.6523 PMC6179001

[6] Storey L, Fern LA, Martins A, Wells M, Bennister L, Gerrand C, et al. A critical review of the impact of Sarcoma on Psychosocial Wellbeing. Sarcoma. 2019;2019:9730867. 30911268 10.1155/2019/9730867 PMC6397984

[7] Flaherty SA. Pain measurement tools for clinical practice and research. AANA J. 1996;64:133–40. 9095685

[8] Card EB, Wells N, Mesko P, Eliades A, MacDonald R, Krenzischek DA. Perianesthesia nurses Pain Management practices: findings and recommendations from a National Descriptive Study of members of the American Society of Perianesthesia Nurses. J Perianesth Nurs. 2021;36:128–35. 33218877 10.1016/j.jopan.2020.07.007

[9] Husted H, Hansen HC, Holm G, Bach-Dal C, Rud K, Andersen KL, et al. What determines length of stay after total hip and knee arthroplasty? A nationwide study in Denmark. Arch Orthop Trauma Surg. 2010;130:263–8. 19633865 10.1007/s00402-009-0940-7

[10] Jensen CB, Troelsen A, Foss NB, Nielsen CS, Lindberg-Larsen M, Gromov K. 10-year evolution of day-case hip and knee arthroplasty: a Danish nationwide register study of 166,833 procedures from 2010 to 2020. Acta Orthop. 2023;94:178–84. 37074191 10.2340/17453674.2023.11961 PMC10116885

[11] Danielsen O, Varnum C, Jensen CB, Jakobsen T, Andersen MR, Bieder MJ, et al. Implementation of outpatient hip and knee arthroplasty in a multicenter public healthcare setting. Acta Orthop. 2024;95:219–24. 38715473 10.2340/17453674.2024.40185 PMC11077343

[12] Constipation After Surgery [Internet]. [cited 2023 Oct 8]; https://www.healthline.com/health/digestive-health/constipation-after-surgery#_noHeaderPrefixedContent

[13] Trads M, Pedersen PU. Constipation and defecation pattern the first 30 days after hip fracture. Int J Nurs Pract. 2015;21:598–604. 24758257 10.1111/ijn.12312

[14] Sharma P, Bhutta BS. Assisting patients with elimination. StatPearls Publishing, Treasure Island (FL). 32644684

[15] Hjalte F, Berggren AC, Bergendahl H, Hjortsberg C. The direct and indirect costs of opioid-induced constipation. J Pain Symptom Manage. 2010;40:696–703. 20727708 10.1016/j.jpainsymman.2010.02.019

[16] Ingrid Poulsen. (f. 1956-12-05); Nina Beyer; illustrator: Birgitte Lerche. Inaktivitet Og Immobilitet - I et tværfagligt perspektiv. 2. Udgave. Munksgaard Danmark.

[17] Morfin. DAK [Internet]. [cited 2023 Oct 2]; https://pro.medicin.dk/Medicin/Praeparater/993

[18] Rao SS, Bharucha AE, Chiarioni G, Felt-Bersma R, Knowles C, Malcolm A et al. Funct Anorectal Disorders Gastroenterol 2016;S0016-5085(16)00175-X. 10.1053/j.gastro.2016.02.009 PMC5035713 27144630

[19] Bulut A, Vatansever NA. Determination of factors affecting early mobilization of patients who have undergone knee and hip arthroplasty. J Perianesth Nurs. 2022;37:646–53. 35525826 10.1016/j.jopan.2021.10.013

[20] Yue C, Liu Y, Zhang X, Xu B, Sheng H. Randomised controlled trial of a comprehensive protocol for preventing constipation following total hip arthroplasty. J Clin Nurs. 2020;29:2863–71. 32320100 10.1111/jocn.15299

[21] Moraitis A, Hultin M, Walldén J. Risk of postoperative nausea and vomiting in hip and knee arthroplasty: a prospective cohort study after spinal anaesthesia including intrathecal morphine. BMC Anesthesiol. 2020;20:242. 32972366 10.1186/s12871-020-01154-z PMC7517815

[22] Fawcett WJ, Jones CN. Bespoke intra-operative anaesthesia - the end of the formulaic approach? Anaesthesia. 2018;73:1062–6. 29533468 10.1111/anae.14253

[23] Macario A, Weinger M, Carney S, Kim A. Which clinical anesthesia outcomes are important to avoid? The perspective of patients. Anesth Analg. 1999;89:652–8. 10475299 10.1097/00000539-199909000-00022

[24] Pinto PR, McIntyre T, Ferrero R, Almeida A, Araújo-Soares V. Risk factors for moderate and severe persistent pain in patients undergoing total knee and hip arthroplasty: a prospective predictive study. PLoS ONE. 2013;8:e73917. 24058502 10.1371/journal.pone.0073917 PMC3772812

[25] Mads U. Werner Og Nanna B. Finnerup Og Lars Arendt-Nielsen (red.). Smerter – 4. Udgave Baggrund, evidens Og behandling. 4th ed. FADL’s Forlag.

[26] Ahmed A, Baig T. Incidence of lower limb motor weakness in patients receiving postoperative epidural analgesia and factors associated with it: An observational study. Saudi J Anaesth [Internet] 2016 [cited 2022 Dec 18];10:149. http://www.saudija.org/text.asp?2016/10/2/149/168806 10.4103/1658-354X.168806 PMC4799605 27051364

[27] Encare ERAS [Internet]. [cited 2023 Sep 25];Available from: https://encare.net/?utm_term=enhanced%20recovery&utm_campaign=USA&utm_source=adwords&utm_medium=ppc&hsa_acc=1250751950&hsa_cam=817649162&hsa_grp=147960338815&hsa_ad=652855166534&hsa_src=g&hsa_tgt=kwd-299558269819&hsa_kw=enhanced%20recovery&hsa_mt=b&hsa_net=adwords&hsa_ver=3&gad=1&gclid=EAIaIQobChMI-L3rmrPW_gIVvhkGAB0sTwjwEAAYASAAEgIpY_D_BwE

[28] Tayrose G, Newman D, Slover J, Jaffe F, Hunter T, Bosco J. Rapid mobilization decreases length-of-stay in joint replacement patients. Bull Hosp Jt Dis (2013) 2013;71:222–6. 24151950

[29] Wylde V, Rooker J, Halliday L, Blom A. Acute postoperative pain at rest after hip and knee arthroplasty: severity, sensory qualities and impact on sleep. Orthop Traumatol Surg Res. 2011;97:139–44. 21388906 10.1016/j.otsr.2010.12.003

[30] Koh SJ, Keam B, Hyun MK, Ju Seo J, Uk Park K, Oh SY, et al. Cancer Pain Management Education rectifies patients’ misconceptions of Cancer Pain, reduces Pain, and improves quality of life. Pain Med. 2018;19:2546–55. 29590446 10.1093/pm/pny039

[31] Ju M, Tao Y, Lu Y, Ding L, Weng X, Wang S, et al. Evaluation of sleep quality in adolescent patients with osteosarcoma using Pittsburgh Sleep Quality Index. Eur J Cancer Care (Engl). 2019;28:e13065. 31012535 10.1111/ecc.13065

[32] Fortmann J, Fisher A, Hough R, Gregory A, Pugh G. Sleep Quality among teenagers and Young adults with Cancer. Cancer Nurs. 2021;44:13–9. 30921031 10.1097/NCC.0000000000000707

[33] Kamdar BB, Needham DM, Collop NA. Sleep deprivation in critical illness: its role in physical and psychological recovery. J Intensive Care Med. 2012;27:97–111. 21220271 10.1177/0885066610394322 PMC3299928

[34] Prophylactic Antibiotic Regimens in Tumor Surgery (PARITY), Investigators, Ghert M, Schneider P, Guyatt G, Thabane L, Vélez R, et al. Comparison of prophylactic intravenous antibiotic regimens after Endoprosthetic Reconstruction for Lower Extremity Bone tumors: a Randomized Clinical Trial. JAMA Oncol. 2022;8:345–53. 34989778 10.1001/jamaoncol.2021.6628 PMC8739829

